# New antimicrobial compounds produced by *Seltsamia galinsogisoli* sp. nov., isolated from *Galinsoga parviflora* as potential inhibitors of FtsZ

**DOI:** 10.1038/s41598-019-44810-2

**Published:** 2019-06-05

**Authors:** Tian-Yuan Zhang, Ying-Ying Wu, Meng-Yue Zhang, Juan Cheng, Blessings Dube, Hui-Jia Yu, Yi-Xuan Zhang

**Affiliations:** School of Life Science and Biopharmaceutics, Shenyang Pharmaceutical University, Shenyang, 110016 China

**Keywords:** Pharmaceutics, Drug development

## Abstract

A total amount of 116 fungal strains, belonging to 30 genera, were acquired from the rhizosphere soil and plant of *Galinsoga parviflora*. A strain SYPF 7336, isolated from the rhizospheric soil, was identified as *Seltsamia galinsogisoli* sp. nov., by morphological and molecular analyses, which displayed high antibacterial activity. In order to study the secondary metabolites of *Seltsamia galinsogisoli* sp. nov., nine compounds were successfully seperated from the strain fermentation broth, including two new compounds and seven known compounds. Their structures were elucidated based on spectral analysis including 1D and 2D NMR. All the seperated compounds were evaluated for their antimicrobial activities. Compounds **2, 5** and **1** displayed antimicrobial activities against *Staphylococcus aureus* with MIC values of 25, 32 and 75 μg/mL, respectively. Moreover, morphological observation showed the coccoid cells of *S. aureus* to be swollen to a volume of 1.4 to 1.7-fold after treatment with compounds **1**, **2** and **5**, respectively. Molecular docking was carried out to investigate interactions of filamentous temperature-sensitive protein Z (FtsZ) with compounds **1**, **2** and **5**.

## Introduction

Secondary metabolites coming from microorganism represent a large number of diverse components which have been treated as potential candidates for drugs^1–5^. Currently, due to the increase of antibiotic resistance, there is an urgent need for novel classes of lead compounds and novel mechanisms to confront the antibiotic crises^6,7^.

Microbial filamentous temperature-sensitive protein Z (FtsZ) is a novel target for drug discovery, which plays a key role in cell division^8,9^. The inhibitors of FtsZ prevent the cellular fission of bacteria, which lead to apoptosis of bacteria^10–12^. Therefore, morphological observation of microbial fission and molecular docking between the lead compound and FtsZ were accomplished to explore the possible mechanism^13,14^. Up to now, the discovered FtsZ inhibitors are divided into natural products (sanguinarine, berberine, totarol, curcumin and cinnamaldehyde) and synthetic small molecules (PC190723, UCM53, CCR-11)^15–19^. At present, PC190723 has been the candidate drugs to enter the clinical trials, which inspires scientists to make more effort to find potential FtsZ inhibitors as antibacterial agents^20–22^.

The endophytic fungi are known as a source of abundant secondary metabolites for functional bioactive substances. As we know, only a small amount of microbes have been studied so far, and a vast number of new taxa waiting for discovery, especially those separated from medicinal plants. This leads us to isolate and evaluate the potential pharmacological activity of bioactive compounds produced by endophytic fungi.

In the present study, the endophytic fungi diversity of the traditional Chinese herb, *Galinsoga parviflora* was surveyed and 116 fungal strains were isolated from the whole plant (stems, leaves and roots) which attributed to 30 genera. Forty-three percent of the strains revealed antimicrobial abilities against at least one kind of human pathogenic microorganisms. Strain SYPF 7336, presented the strongest antibacterial activity, could not be affiliated to any known taxon, so it was identified as a novel species of genus *Seltsamia* by phylogenetic analyses, given the name *Seltsamia galinsogisoli* sp. nov. The genus *Seltsamia*, (*Cucurbitariaceae*) was first proposed in 2018, and the only strain *Seltsamia ulmi* CBS 143002 was isolated on corticated *Ulmus glabra* in Norway^23^. *Seltsamia ulmi* produces pyriform, black ascomata and cylindrical asci. Asci contain 8 uni- to partly biseriately arranged ascospores and ascospores are fusoid to subclavate with 3 main septa. Due to none of the secondary metabolites of the genus *Seltsamia* having been recorded, the compounds and bioactivities of the novel species, strain SYPF 7336, were explored in the present study. Further, the molecular docking analysis was carried out for mechanism investigation.

## Results

### Species delineation and classification

**Figure 1:**
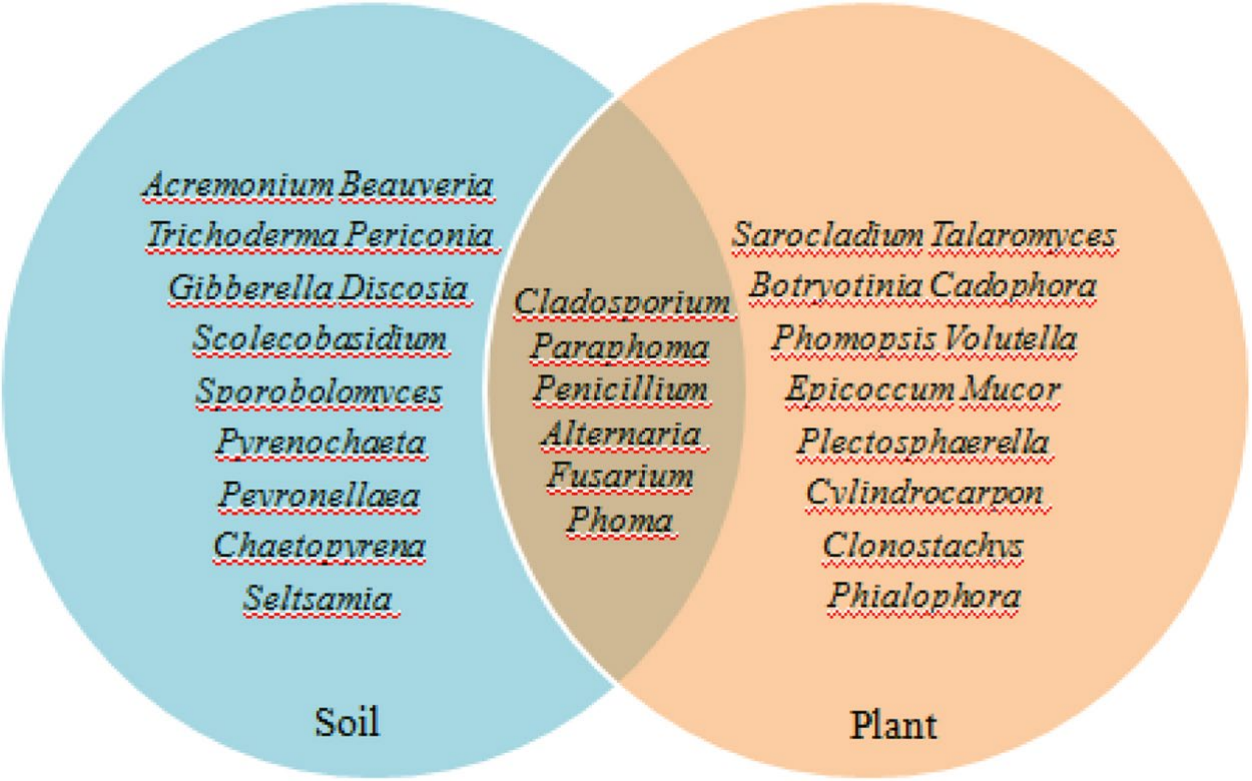
Genus distribution of endophytic fungi isolated from soil and plant of *Galinsoga parviflora*.

A total of 116 fungal strains were acquired from the soil and plant of *Galinsoga parviflora*, which were divided into 30 groups according to morphological characteristics and phylogenetic analyses. Twelve genera, *Acremonium, Beauveria, Trichoderma, Periconia, Gibberella, Discosi*, *Scolecobasidium*, *Sporobolomyces*, *Pyrenochaeta*, *Peyronellaea*, *Chaetopyrena* and *Seltsamia*, were found only in the soil. Twelve genera, *Sarocladium*, *Talaromyces*, *Botryotinia*, *Cadophora*, *Phomopsis*, *Volutella, Epicoccum*, *Mucor, Plectosphaerella, Cylindrocarpon*, *Clonostachys*, and *Phialophora* just survived in the plant. Six genera, *Cladosporium, Paraphoma, Penicillium, Alternaria, Fusarium*, and *Phoma* could simultaneously survive in the soil and plant of *G. parviflora* (Fig. 1).

**Figure 2:**
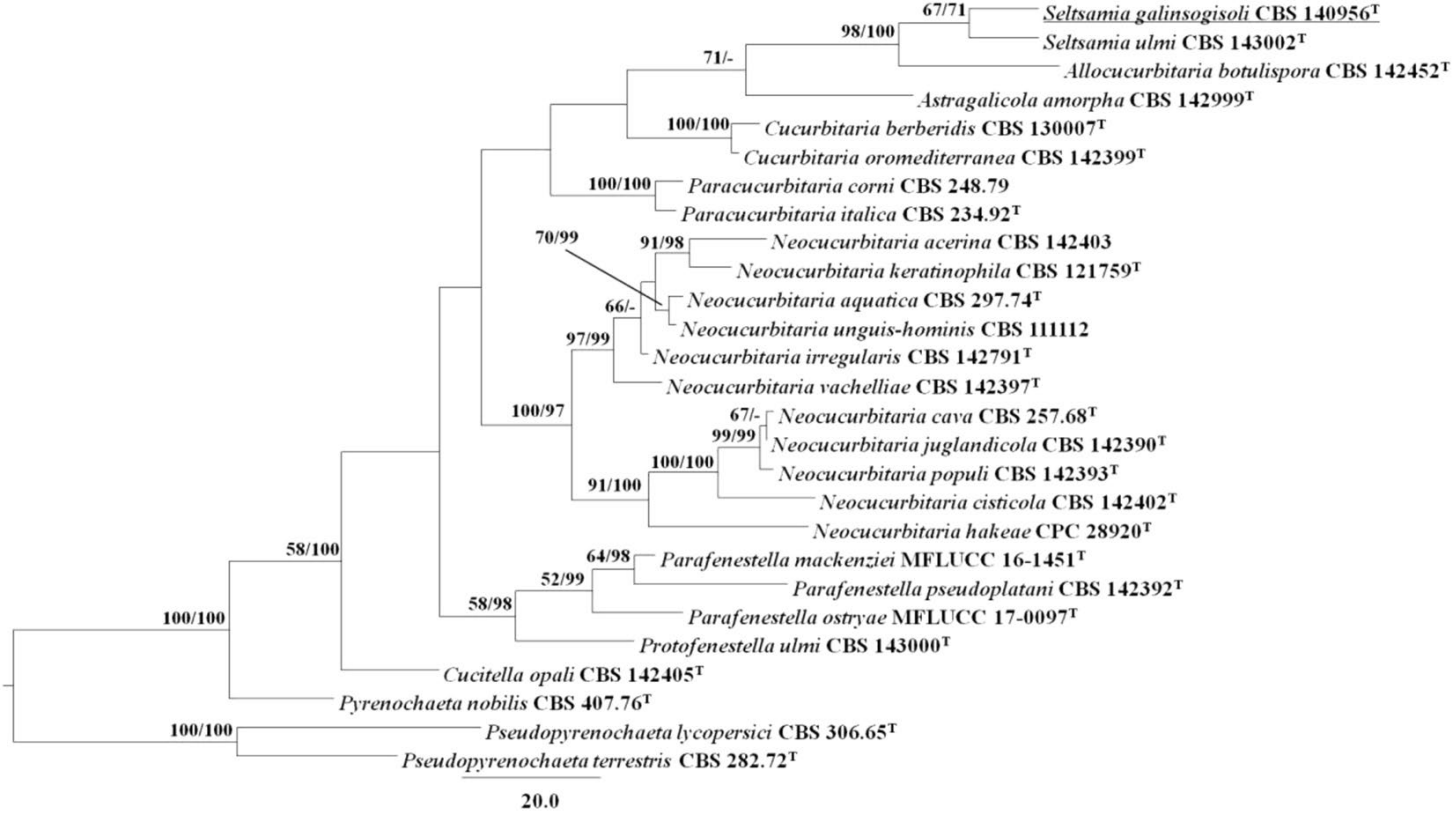
Maximum Parsimony (MP) tree based on analysis of a combined dataset of ITS and LSU sequence data. MP bootstrap support values (MPB above 50%) and Bayesian posterior probabilities (BPP; above 70%) are given at the nodes (MPB/BPP). *Drechmeria panacis* sp. nov. is denoted by bold letters.

A strain SYPF 7336, isolated from the rhizosphere soil of *G. parviflora*, was most close to *Seltsamia ulmi* when using ITS sequence BLAST, but they did not match well in 13 different positions. So, the phylogenetic analyses were carried out based on two loci (ITS and LSU) to clarify the taxon status of strain SYPF 7336. In the MP analysis, 1938 characters were constant, 87 were parsimony-uninformative, and 152 were parsimony-informative. After phylogenetic analysis, the best MP tree was shown in Fig. 2 (TL = 576, CI = 0.575, RI = 0.630, RC = 0.362, HI = 0.425). In this tree, strain SYPF 7336 was placed in genus *Seltsamia* and formed a sister clade together with *S. ulmi*. The ITS and LSU sequences were deposited in GenBank with accession numbers KU759584 and KU759581, respectively (Table 1).

**Table 1:** Strains used in the phylogenetic analyses and their corresponding GenBank accession numbers.

Species	Culture accession No.	GenBank accession No.	
		ITS	LSU
*Allocucurbitaria botulispora*	CBS 142452^T^	LT592932	LN907416
*Astragalicola amorpha*	CBS 142999^T^	MF795753	MF795753
*Cucitella opali*	CBS 142405^T^	MF795754	MF795754
*Cucurbitaria berberidis*	CBS 130007^T^	LT717673	KC506793
*Cucurbitaria oromediterranea*	CBS 142399^T^	MF795761	MF795761
*Neocucurbitaria acerina*	CBS 142403	MF795768	MF795768
*Neocucurbitaria aquatica*	CBS 297.74^T^	LT623221	EU754177
*Neocucurbitaria cava*	CBS 257.68^T^	JF740260	EU754199
*Neocucurbitaria cisticola*	CBS 142402^T^	MF795772	MF795772
*Neocucurbitaria hakeae*	CPC 28920^T^	KY173436	KY173526
*Neocucurbitaria irregularis*	CBS 142791^T^	LT592916	LN907372
*Neocucurbitaria juglandicola*	CBS 142390^T^	MF795773	MF795773
*Neocucurbitaria keratinophila*	CBS 121759^T^	EU885415	LT623215
*Neocucurbitaria populi*	CBS 142393^T^	MF795774	MF795774
*Neocucurbitaria unguis-hominis*	CBS 111112	LT623222	GQ387623
*Neocucurbitaria vachelliae*	CBS 142397^T^	MF795787	MF795787
*Paracucurbitaria corni*	CBS 248.79	LT903672	GQ387608
*Paracucurbitaria italica*	CBS 234.92^T^	LT623219	EU754176
*Parafenestella mackenziei*	MFLUCC 16-145^T^	KY563071	KY563074
*Parafenestella ostryae*	MFLUCC 17-0097^T^	KY563072	KY563075
*Parafenestella pseudoplatani*	C BS 142392^T^	MF795788	MF795788
*Protofenestella ulmi*	CBS 143000^T^	MF795791	MF795791
*Pseudopyrenochaeta lycopersici*	CBS 306.65^T^	NR_103581	EU754205
*Pseudopyrenochaeta terrestris*	CBS 282.72^T^	LT623228	LT623216
*Pyrenochaeta nobilis*	CBS 407.76^T^	EU930011	EU754206
***Seltsamia galinsogisoli*** ^a^	**CBS 140956** ^T^	**KU759584**	**KU759581**
*Seltsamia ulmi*	CBS 143002^T^	MF795794	MF795794

“^a^”New accession numbers produced in this study are bold.

### Description of Seltsamia galinsogisoli Tianyuan Zhang & Yixuan Zhang, sp. nov. MB 820393

*Seltsamia galinsogisoli* (Ga.lin.so.gi’so’li. N.L. gen. n. *galinsogisoli* of soil of a *G. parviflora*, got from Huludao city, Liaoning Province, northeast of China).

**Figure 3:**
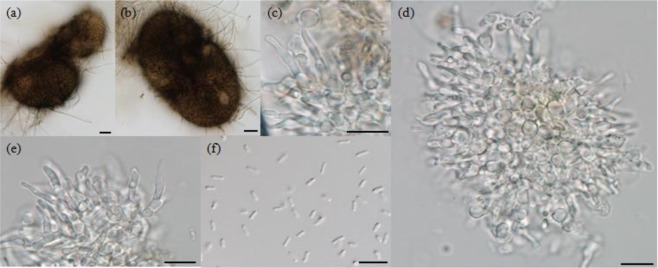
Morphological characters of *Seltsamia galinsogisoli* CBS 140956^T^. (**a**,**b**) Pycnidia. (**c**–**e**) Conidiogenous cells. (**f**) Conidia. Bars: **a**,**b** = 20 μm, **c**–**f** = 10 μm.

Vegetative hyphae hyaline, smooth walled. Pycnidia subglobose to globose, brown to dark brown, 70–125 × 45–96 μm. Surface roughened by colourless hyphal appendages (Fig. 3a,b). Conidiogenous cells phialidic, hyaline, smooth walled, 9–19.2 × 1.4–4.2 μm (Fig. 3c–e). Conidia 1-celled, hyaline, smooth, cylindrical, slightly curved, 2.5–4.3 × 0.8–1.2 μm (Fig. 3f).

**Figure 4:**
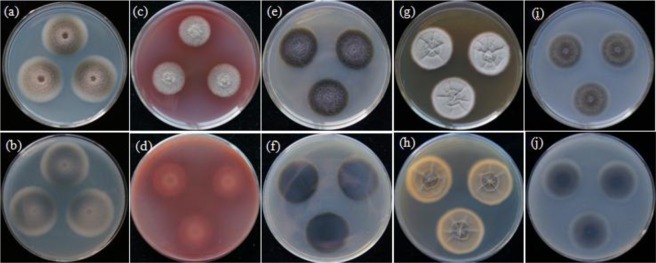
Colony morphologies of *Seltsamia galinsogisoli* CBS 140956^T^ (**a**–**j**). Colonies on PDA at 26 °C after 11 d (**a**, obverse; **b**, reverse), on PNA at 26 °C after 11 d (**c**, obverse; **d**, reverse), on CMA at 26 °C after 11 d (**e**, obverse; **f**, reverse), on MEA at 26 °C after 24 d (**g**, obverse; **h**, reverse) and on OA at 26 °C after 11 d (**i**, obverse; **j**, reverse).

Colonies on PDA attaining 36.6 mm diameter after 11 d at 26 °C, surface floccose, grey to dark grey, reverse grey to dark grey (Fig. 4a,b). Colonies on PNA attaining 25.9 mm diameter after11 d at 26 °C, surface floccose, grey, reverse grey (Fig. 4c,d). Colonies on CMA attaining 30.2 mm diameter after 11 d at 26 °C, surface floccose, dark grey to dark green, reverse dark green to black (Fig. 4e,f). Colonies on MEA attaining 33.5 mm diameter after 11 d at 26 °C, surface velvety, grey with 5–8 radial and 1 annular groove, reverse grey with 5–8 radial and 1 annular cracks (Fig. 4g,h).Colonies on OA attaining 29.8 mm diameter after 11 d at 26 °C, surface floccose, grey to dark olive green, reverse dark olive green (Fig. 4i,j).

#### Type specimen

China, Liaoning province, Huludao city, 40°82′26.5″N, 119°78′52.0″E, Sep 2014, from the rhizosphere of *G. parviflora*. Ex-type culture CBS 140956 = CGMCC 3.17981 = SYPF 7336.

### Identification of the compounds

**Figure 5:**
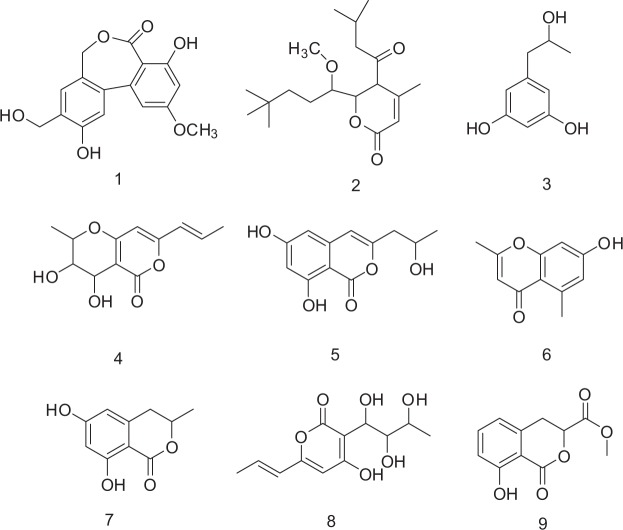
Chemical structures of compounds **1–9**.

Seltsamiayu (**1**) (Fig. 5) was isolated as white flakes. The absorption bands at 3448 (strong wide wave), 1636.6 cm^−1^ in the IR spectrum suggested the presence of hydroxyl carboxylic acid carbonyl, and carbonyl functionalities (Figs S1–S6). The molecular formula of compound **1** was determined as C_16_H_14_O_6_ on the basis of its ion [M + Na]^+^ at *m/z* 325.2738 obtained by HRESI/MS.

**Figure 6:**
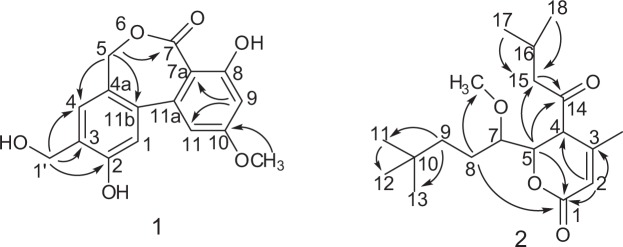
Selected HMBC correlations of compounds **1** and **2**.

The 1D NMR spectra of compound **1** (Table 2) indicated the presence of two benzene rings [*δ*_C_ 162.2, 159.9, 146.6, 145.9, 140.1, 129.8, 126.7, 115.6, 115.5, 109.5, 105.0, 100.8; *δ*_H_ 7.04 s, 6.90 s, 6.51 d (*J* = 2.2 Hz),6.50 d (*J* = 2.2 Hz)] and one methoxy [*δ*_C_ 55.4,*δ*_H_ 3.8 s]. The spectra of **1** were in part very similar to those of alter lactone except for the absence of a methylene group^24^. The HMBC spectra indicated the presence of long-range correlations (Fig. 6) from H-1′ with C-2, C-3, and C-4, from H-5 with C-4a, C-7 and C-11b, from H-9 with C-7a and C-11, combined with the HSQC and HRESIMS data, the structure of compound **1** was verified as shown in Fig. 5 and it was given the common name Pyrenochaetayu.

**Table 2:** ^1^H (600 MHz, DMSO-*d*_4_) and ^13^C NMR (150 MHz, DMSO-*d*_4_) data of compounds **1** and **2**.

Compd. No	1		Compd. No.	2	
	*δ*C, type	*δ*H (*J*, Hz)		*δ*C, type	*δ*H (*J*, Hz)
1	115.5	7.04 (s, 1H)	1	174.3, C	—
2	146.6	—	2	116.3, CH	5.85 (s, 1H)
3	145.9	—	3	151.2, C	—
4	115.6	6.90 (s,1H)	4	103.5, C	—
4a	140.1	—	5	87.7, CH	4.83 (m,1H)
5	67.8	4.86 (d, 1H, *J* = 11.0) 4.85 (d, 1H, *J* = 11.0)	6	O	—
7	168.7	—	7	51.9, CH	3.6 (m, 1H)
7a	109.5	—	7-OCH3	58.9, CH_3_	3.68 (m, 3H)
8	159.9	—	8	46.4, CH_2_	4.05 (m, 2H)
9	100.8	6.51 (d, 1H, *J* = 2.2)	9	32.4, CH_2_	2.66 (m, 2H)
10	162.2	—	10	29.1, C(丟)	—
11	105.0	6.50 (d, 1H, *J* = 2.2)	12-CH_3_	20.7, CH_3_	1.59 (m, 3H, *J* = 7.3)
11a	126.7	—	11-CH_3_	8.2, CH_3_	0.92 (t, 3H, *J* = 7.3)
11b	129.8	—	13-CH_3_	13.4, CH_3_	0.97 (t, 3H, *J* = 7.3)
10-OCH_3_	55.4	3.8 (s, 3H)	14	193.0, C	—
3-CH_2_-OH	48.6	4.83 (dd, 2H, *J* = 11.15)	15	23.5, CH_2_	1.95 (m,1H) 1.75 (m,1H)
2-OH	—	9.39	16	45.6, CH	2.92 (brs, 1H)
3-CH_2_-OH	—	9.46	17	22.2, CH_3_	0.849 (m, 3H)
8-OH	—	10.2	18	22.2, CH_3_	0.877 (m, 3H)

Galinsogisoliyu (**2**) was seperated as a brown powder, ${[\alpha ]}_{{\rm{D}}}^{20}$ + 3.7 (c 0.25, MeOH). The absorption bands at 3443 (strong wide wave),1703 and 1642 cm^−1^ in IR spectrum suggested the presence of hydroxyl carboxylic acid carbonyl, and carbonyl functionalities (Figs S7–12). The HRESIMS indicated the presence of an ion peak at m/z 347.4509 [M + Na]^+^ (calcd. for C_19_H_32_O_4_Na, 347.4504), indicating the molecular formula of C_19_H_32_O_4_. The ^1^H NMR spectrum of **2** (Table 2) indicated the presence of five methyl groups at *δ*_H_ 0.92(t, 3H), 0.97 (t, 3H), 0.849 (m, 3H), 0.877 (m, 3H), 1.59 (m, 3H), one methoxyl groups at *δ*_H_ 3.68 (3H, m) [Henrick *et al*. 1975, Watanabe *et al*. 2000]. It also showed four methylene protons at *δ*_H_ 4.05 (2H, m, H-8), 2.66 (2H, m, H-9), 1.95 (m, 1H, H-15a) and 1.75 (m, 1H, H-15b), five methine protons at 5.85 (1H, s, H-2), 4.83 (1H, m, H-5), 3.6 (1H, m, H-7), 3.6 (1H, m, H-7), 2.92 (1H, brs, H-16); The ^13^C NMR spectra (Table 2) indicated the presence of two carbonyl carbons (*δ*_C_ 174.3 and 193.0), a pair of olefinic carbons (*δ*_C_ 174.3 and 193.0), five methyl carbons (*δ*_C_ 22.2*2, 20.7, 13.4 and 8.2), one oxygenated methylene proton carbon (*δ*_C_ 58.9), three methylene carbons (*δ*_C_ 46.4, 32.4 and 23.5). The ^1^H and ^13^C NMR spectra of **2** were similar to those of 4-methyl-5,6-dihydro-2H-pyran-2-one except for the absence of two side chains^25,26^. The planar structure of 2 (Fig. 6) was established by the 2D NMR data. The 2D NMR spectra of compound 2 (Fig. 6) indicated the presence of long-range correlations from H-2 with C-1, C-3 and C-4, H-5 with C-1, C-14 and C-15, H-9 with C-11 and C-13 and H-15 with C-5 and C-14. According to the above evidence, the structure of **2** was verified as shown in Fig. 5 and it was given the common name Galinsogisoliyu.

Additionally, the discovery metabolites of *Seltsamia galinsogisoli* sp.nov. resulted the isolation of seven known compounds (**3–9**), including 1,3-Benzenediol,5-(2-hydroxypropyl) (**3**)^27^, 3,4-Dihydroxy-2-methyl-7-[prop-1-enyl]-3,4-dihydro-2H-pyrano[4,3-b]pyran-5-one (**4**)^28–30^, 1H-2-Benzopyran-1-one,6,8-dihydroxy-3-(2-hydroxypropyl) (**5**)^31,32^, 2,5-dimethyl-7-hydroxyl chromone (**6**)^33^, 3-methyl-6,8-dihydroxyisocoumarin (**7**)^34^, Curvulapyrone (**8**)^34^, and 3,4-dihydro-8-hydroxyisocoumarin-3-carboxylic methyl ether (**9**)^35^.

### The antimicrobial results of compounds 1–9

All the seperated metabolites were tested for antimicrobial effects against five common pathogenic bacteria, *S. aureus*, *B. subtilis*, *P. aeruginosa*, *K. pneumonia* and *Bacillus cereus*. The results were shown in Table 3.

**Table 3:** Antibacterial effects of compounds **1–9**.

Compd.	*Staphylococcus aureus*	*Bacillus subtillis*	*Pseudomonas aeruginosa*	*Klebsiella pneumonia*	*Bacillus cereus*
**1**	+++	++	++	+	+
**2**	+++	+	+	+	+
**3**	++	+++	−	++	++
**4**	+	+++	+	+	++
**5**	+++	+++	++	+	++
**6**	++	+	+	+	++
**7**	++	++	++	+	++
**8**	++	+++	−	+	++
**9**	+	−	+	+	+
Ampicillin^a^	++++	++++	++++	++++	++++

^*a*^As positive control. “+”: antibacterial rate 0–30%, “+”: 30–60%, “+++”, 60–80%, “++++”, 80–100%, “−” no antibacterial activities.

Compounds **2, 5** and **1** showed antimicrobial activities against *S. aureus* with MIC values of 25, 32 and 75 μg/mL, respectively. Compounds **3–4**, **6–7** and **8–9** showed weak antimicrobial effects.

### Morphological observation and molecular docking

**Figure 7:**
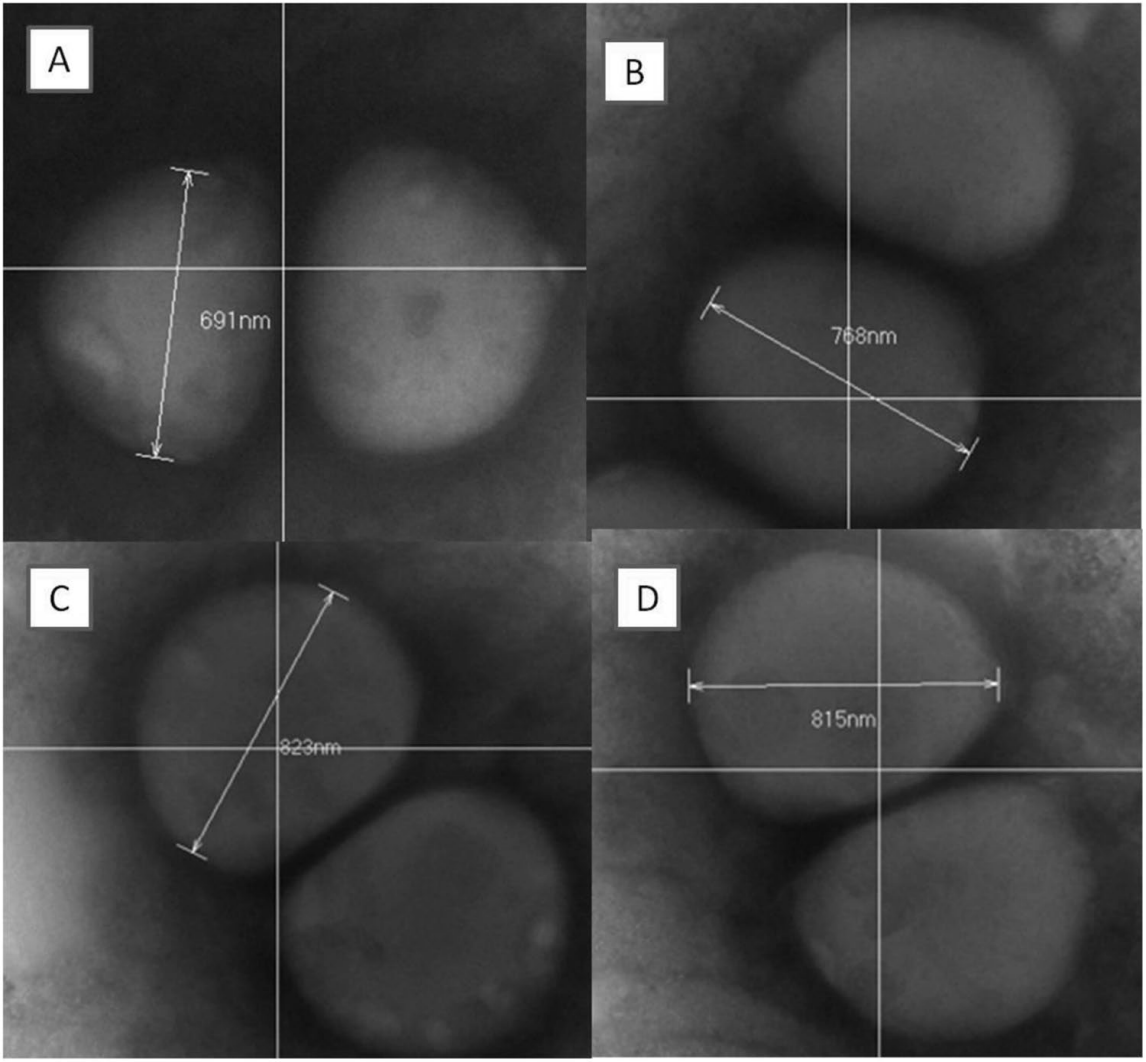
Electron micrographs of *S. aureus* in the absence (**A**) or presence of compounds 1 (**B**), 2 (**C**) and 5 (**D**). (**A**) Untreated control cells of *S. aureus* with average diameter length of 691 nm. (**B**) cells of *S. aureus* with average diameter length of 768 nm. (**C**) Cells of *S. aureus* with average diameter length of 823 nm. (**D**) Cells of *S. aureus* with average diameter length of 815 nm.

The cells of *S. aureus* treated with compounds were observed carefully (Fig. 7). Interestingly, the coccoid cells of *S. aureus* were swelled to larger volume after treatment with compound **1** (1.4 fold), **2** (1.7 fold) and **5** (1.6 fold), respectively. In order to explain the possible mechanism, FtsZ, key protein of cell division^35^, was explored for molecular docking study.

**Figure 8:**
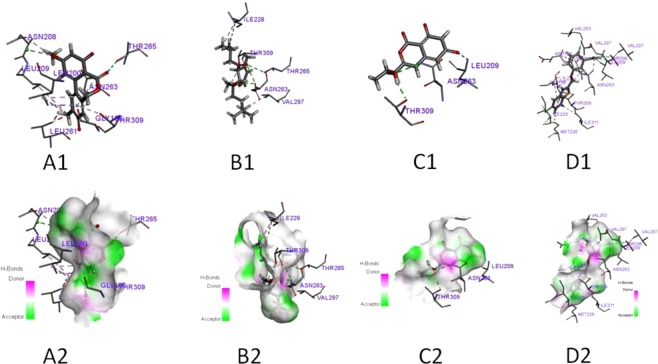
In silico docking simulation of compounds **1, 2, 5** and ligand to FtsZ of *S. aureus*. (**A**_**1**_–**D**_**1**_) H-bond interactions between **1, 2, 5** and ligand to FtsZ of *S. aureus*. (**A**_**2**_–**D**_**2**_) Hydrophobic interaction between **1, 2, 5** and ligand to FtsZ of *S. aureus* in a 3D docking model.

The docking simulation of active compounds **1**, **2** and **5** to FtsZ from *S. aureus* (PDB:ID 3VOB) (Fig. 8) resulted in the binding energies of −109, −125, and −113 kcal/mol, respectively (Table 4). Thus, compound **2** had the best binding energies with FtsZ. Furthermore, the binding patterns were also different. Compound **2** displayed four hydrogen bonds and one more hydrophobic bond to relevant residues comparing with compound **5** (Table 4). Compound **1** showed two hydrogen bonds and five hydrophobic bonds to relevant residues. However, an unfavorable bump LEU261 was observed for compound **1** (Table 4). Five hydrophobic bonds for compound **1** were observed and considered to make major contribution to the combinations. The interactions between FtsZ with compounds **1–2** and **5** are displayed in Table 4.

**Table 4:** Binding residues involved in the formation of hydrophobic bonds and hydrogen bonds with compounds **1, 2, 5** and ligand.

Comd.	Docking score (Kcal/mol)	Unfavorable Bump	Residues	
			Conventional hydrogen bond	Hydrophobic bond
**1**	−109	LEU261	LEU209, LEU200	ASN208, THR309, ASN263, GLY196,THR265
**2**	−125	—	ILE228*2, THR309, VAL297	ASN263*2,THR309, THR265
**5**	−113	—	—	THR309, ASN263, LEU209
ligand	−167	—	LEU209, ASN 263, THR 296, VAL 207, GLY 196, THR 309, VAL 203	ILE 228, ILE311, ILE 197, LEU200, ASP 199, VAL 297, MET 226

“—” No bond connected “*” Multiple bond connected.

## Discussion

All the endophytic fungi isolated from the rhizosphere of *G. parviflora* were fermented and the crude extracts of each strain were tested for the microbial activities (Table S1). Forty-three percent of the strains showed antimicrobial activities against at least one kind of human pathogenic microorganisms. These results provide references for further study of the strains.

Among the stains, SYPF 7336, showed the best antibacterial activity. The strain SYPF 7336 was carefully studied and identified as *Seltsamia galinsogisoli* sp. nov. by morphology and molecular analyses, and only pycnidia was observed whereas no perfect stage available. This is the first-found of pycnidia in the genus *Seltsamia* for only perfect stage was recorded whereas no asexual information in the publication in *S. ulmi*, which was isolated from *Hapalocystis bicaudata* on corticated *Ulmus glabra* in Norway in 2018^23^. Though differences of the reproductive body between the two species could not be compared, they are remarkably different species based on the phylogenetic analyses (Fig. 2). Moreover, the difference between the two species are that *Seltsamia galinsogisoli* sp. nov. produces dark grey **c**olonies with annular and radical grooves on MEA^23^. *Seltsamia* is a newly introduced genus, the finding of *Seltsamia galinsogisoli* sp. nov. expands the host range of this genus.

Another aim of this study is to isolate antimicrobial secondary metabolites secreted by the novel strain, *Seltsamia galinsogisoli* sp. nov. It is the first time to report secondary metabolites from the genus *Seltsamia*, family *Cucurbitariaceae*. Two new compounds (**1–2**) and seven known compounds (**3–9**) (Fig. 5) were purified, identified and tested for their antimicrobial abilities against *S. aureus*, *B. subtilis*, *P. aeruginosa*, *K. pneumonia*, and *E. coli*. As results, compounds **2, 5** and **1** displayed well antibacterial activities toward *S. aureus* with MIC values of 25, 32 and 75 μg/mL, respectively. These results from the present work provide further information about the diversity and activities of compounds in the genus *Seltsamai*.

FtsZ is a pop target for drug discovery in recent years. The gene of FtsZ has the ability of high conservation and presented almost in all bacteria^9,36^. In bacterial cytokinesis, FtsZ protein is the earliest known step to build a contractile ring on the inner surface of the cytoplasmic membrane^9,36^. The inhibitors of FtsZ might prevent the cellular fission of bacteria, which lead to apoptosis of bacteria. Therefore, morphological observation and molecular docking were carried out to search the possible interactions between the active compounds and FtsZ.

Thus, the cells of *S. aureus* treated with active compounds **1**, **2** and **5** were observed to further study the possible antibacterial mechanism. Interestingly, As Fig. 7 showed, the coccoid cells of *S. aureus* were swollen to 1.4 to 1.7-fold volume after treatment with compound **1** (1.4 fold), **2** (1.7 fold) and **5** (1.6 fold), respectively. In order to explain this interesting appearance, FtsZ, the key protein of cell division^8,9^, was explored to illustrate the mechanism of cells that became swollen. Thus, a molecular docking study was carried out to verify the deduction.

The docking results are shown in Fig. 8 and Table 4. The FtsZ from *S. aureus* (PDB:ID 3VOB) displayed the docking score of the ligand (−167 kcal/mol) was the lowest. Compound **5** (−113 kcal/mol) displayed three hydrophobic bonds with THR309, ASN263, LEU209 residues but no hydrogen bonds with neighbouring amino acid residues. Compound **2** (−125 kcal/mol) formed four hydrogen bonds with the ILE228*2, THR309, VAL297 residues and four hydrophobic bonds with the ASN263*2,THR309 and THR265 residues. Although compound **1** formed two hydrogen bonds and five hydrophobic bonds with the residues, one unfavorable interactions between compound **1** and the active region or intramolecular of FtsZ were existed. Thus, compound **1** showed a weak ability to combine with the docking score of −109 kcal/mol. The docking scores −125 kcal/mol (**2**) −113 kcal/mol (**5**) and -109 kcal/mol (**1**), indicated that compounds **2** and **5** might form lower potential energies and more stable binding sites with the target protein FtsZ compared to coumpound **1** which validated the observed antimicrobial activities. Based on the antimicrobial activities, phenotypic consequences and docking studies, compounds **2** and **5** were identified as promising antimicrobial lead molecules.

## Methods

### General experimental procedures

Optical rotations were recorded using a P-2000 Digital Polarimeter (JASCO, United Kingdom)^37^. IR spectra were measured on an Equinox55 spectrophotometer in KBr discs (Bruker Optik BmbH, Ettlingen, Germany). The 1D- and 2D-NMR spectra were recorded at 600 for ^1^H and 150 MHz for ^13^C (Bruker, Rheinstetten, Germany). HR-ESI-MS data were acquired on a Bruker Customer micrOTOF-Q 125 mass spectrometer (MA, Germany). Solvents were purchased from Tianjin Kemiou Chemical Reagent Company (Tianjin, China), MeOH and CH_3_CN for HPLC analysis were chromatographic grades (Merck, Darmstadt, Germany). Silica gel (200–300 mesh, Qingdao Marine Chemistry Ltd, Qingdao, China) were used for column chromatography.

### Sampling, fungal isolation, morphological study

Samples of soil and plant were collected from the field of a traditional Chinese medical herb *G. parviflora* in Huludao city, Liaoning province, northeast of China (40°82′26.5″N, 119°78′52.0″E). The samples were conducted as described previously^38,39^. All plates were incubated at 26 °C and examined daily. Single colonies were picked and transferred to freshly prepared PDA plates. The single spore-origin strain was stored at 4 °C and/or conidia suspension in 20% glycerol for further study.

The colony morphology of the isolate was studied on PDA, pine-needle agar (PNA), corn meal agar (CMA), malt extract agar (MEA) and oatmeal agar (OA) plates and incubated at 26 °C^37^. Mycelium structure was observed under an optical microscope Olympus BX53 (Olympus, Tokyo, Japan).

### DNA isolation, PCR and sequencing

The mycelia grown in PDB at 26 °C for 7 days were prepared for DNA isolation. Total genomic DNA was extracted as described previously^40,41^. The internal transcribed spacer (ITS) region was amplified with primers ITS4 and ITS5^42^. The partial 28S ribosomal RNA (LSU) gene region was amplified with primers LROR and LR7^43,44^. The thermocycling conditions for amplifications had an initial denaturing step of 94 °C for 5 min, 32 cycles of 94 °C for 60 s, 55 °C for 30 s, 72 °C for 90 s, followed by a final elongation step at 72 °C for 7 min. A G1000 Thermal Cycler (BIOER, Hangzhou, China) was used for PCR amplification. Amplicons were verified with 1% agarose electrophoresis gel, and the expected bands were excised and purified with an AxyPrepTM gel purification kit (Axygen, Hangzhou, China). These fragments were cloned into the pEASY-T5 zero cloning kit (Transgen Biotech, Beijing, China) and followed by sequencing (Sangon Biotech, Shanghai, China). The nucleotide sequences of the genes have been deposited in GenBank (Table 1).

### Phylogenetic analyses

All the sequences (Table 1) were aligned by Clustal X (Larkin, Blackshields *et al*. 2007) and Mega 7.0^45,46^. Datasets were analyzed using both maximum parsimony (MP) and Bayesian tree inference (BI). MP analyses were performed using PAUP* 4.0b10, a heuristic search option was chosen with random addition of sequences as 1,000 replications; gaps were treated as missing data^47^. BI analyses were run with MrBayes 3.2.4 using the GTR substitution model with gamma-distributed rate variation across sites and a proportion of invariable sites^48^. Two sets of four chains were executed until the standard deviation of split frequencies reached 0.01. Sample frequency was set at 100 and 25% of trees removed as burn-in.

### Fermentation and extraction

This assay was performed according to our previous method^37^. Briefy, sterile water (53 ml) and rice (40 g) were mixed to an Erlenmeyer flask (250 ml), which were autoclaved at 121 °C for 30 min. The strain of SYPF 7336 (1 ml) was inoculated in each Erlenmeyer flask (250 ml × 120), which were cultivated at 28 °C for 30 days. The fermented material was extracted using ethyl acetate (12 L × 3) to give the crude extract (127 g). Then it was dissolved in 90% MeOH–H_2_O (1 L), and extracted by hexane (1 L × 3) to obtain the residue (62 g).

### Isolation of secondary metabolites

Silica gel chromatography was used to separate the extract (41.44 g) eluting with CH_2_Cl_2_/CH_3_OH (v/v 80:1–3:1), and yielding four fractions (A-D). Fraction B (10.2 g) was separated by ODS eluting with MeOH-H_2_O (v/v 10:90 to 90:10) to give another eight fractions (B1-B7). Fraction B1 (1.6 g) was further subjected to semipreparative HPLC [Agilent 1100 instrument; YMC 5 µm C18 column (250 mm × 10 mm)], eluted with CH_3_CN-H_2_O (v/v 18:82, 3.5 ml/min) to yield compounds **1** (10 mg), **3** (12 mg) and **8** (10 mg). Subfraction B 3–4 (0.8 g) was further purified by semipreparative HPLC (CH_3_CN-H_2_O, v/v 20:80, 3.5 ml/min) to produce compounds **2** (10 mg), **4** (13 mg) and **9** (11 mg). Similarly, subfraction B7 (1.2 g) was subjected to semipreparative HPLC, eluted with CH_3_CN-H_2_O (v/v 22:78, 3.5 ml/min) to afford compounds **5** (13.6 mg), **6** (11.2 mg) and **7** (17 mg).

Compound **1**: white flakes; ^1^H (600 MHz, DMSO-*d*_6_) and ^13^C NMR (150 MHz, DMSO-*d*_6_) data, see Table 2; HRESIMS *m/z* 325.2738 [M + Na]^+^ (calcd for C_16_H_14_O_6_ Na, 325.2733).

Compound **2**: yellowish oil; ${[\alpha ]}_{{\rm{D}}}^{20}$ − 3.0 (c 0.25, MeOH). ^1^H (600 MHz, DMSO-*d*_6_) and ^13^C NMR (150 MHz, DMSO-*d*_6_) data, see Table 2; HRESIMS *m/z* 307.2551 [M + Na]^+^ (calcd for C_13_H_16_O_7_Na, 307.2556).

### Antimicrobial assay

Bacterial strains used in our anti-bacterial studies are from National Center for Medical Culture Collections (CMCC), and the CMCC numbers are listed as below: *B. subtilis* (CMCC63501), *S. aureus* (CMCC26003), *P. aeruginosa* (CMCC10104), *K. pneumonia* (CMCC46117), and *E. coli* (CMCC44102)^49,50^. The antimicrobial assay was performed according to the procedure of Zhu *et al*.^50^. Briefly, DMSO was used to dissolved the positive control and compounds **1**–**9** at an initial concentration of 10 mg/ml, respectively. Luria-Bertani (LB) media was used for the five pathogenic bacteria for 24 h at 37 °C until the absorbance OD_600_ = 0.2. The bacterial solution was diluted with LB medium (4%). 2 μl sample solution and 198 μl bacterial culture were mixed into 96-well plate. The 96-well plate was determined by a microplate reader (OD_600_) after 24 h of cultivation at 37 °C. Growth inhibition (%) was calculated as [1 − (OD_600_ of treatment/OD_600_ of control)] × 100. Every experiment was performed in triplicate to validate the biological activities.

Minimal inhibitory concentration (MIC) of compounds **1**, **2** and **5** were assessed against bacteria *S. aureus* (CMCC26003). The MIC values of the isolated compounds against human pathogenic bacteria were determined by the modified CLSI M38-A method^49,50^. Briefly, compounds **1**, **2** and **5** were dissolved using DMSO with the final concentrations of 100, 50, 25, 12.5, and 6.25 ug/mL, respectivly for the MIC determination. The dosage-response curve was drawed according to different concentrations of compound to *S. aureus* cells growth inhibition rates. The MIC values were calculated from the dosage-response curves. DMSO and Ampicillin were used as the negative control and positive control, respectively.

### Molecular docking

This assay was performed according to our previous method^37^. Briefy, the crystal structure of protein obtained from RCSB Protein Data Bank (PDB Code 3VOB) were used for docking^51^. The 3D structures of the compounds **1–2** and **5** were prepared and Gasteiger-Hückel charges were added using Sybyl software (Tripos, America). The ligand, guanosine-5′-diphosphate, was subjected to energy minimization with Tripos force filed parameters^51^. Blind docking was carried out using Molegro Virtual Docker 4.0 (Molegro ApS, Aarhus, Denmark) program. The 3D docking grid was sufficiently large to cover the protein.

### Morphological observation of bacterial fission

To identify whether there are changes in morphology of *S. aureus* after treated with compounds **1–2** and **5**, observations under a transmission electron microscope (TEM, HT7700, Japan) were performed. *S. aureus* cells were grown at 37 °C on an agar plate, then diluted by LB broth to an OD_600_ of 0.2, and 25 μg/ml compounds **1–2** and **5** were added to the suspensions. Samples of treated cells and controls were further cultivated at 37 °C for 3 h. Then, the bacterial suspensions were dyed with 2% phosphotungstic acid (v/v = 1:1 pH 6.5) for 3–5 min, and transmission scan was performed as previously described^20^.

## Supplementary information

Supporting information
