# Supplementary material for: New antimicrobial compounds produced by Seltsamia galinsogisoli sp. nov., isolated from Galinsoga parviflora as potential inhibitors of FtsZ
Source: Sci Rep. 2019 Jun 5;9:8319. doi: 10.1038/s41598-019-44810-2 (PMC6549247; doi:10.1038/s41598-019-44810-2)
Supplement: Supplementary file 1 — Supporting information [file 41598_2019_44810_MOESM1_ESM.doc]

**SUPPORTING INFORMATION**

**New antimicrobial compounds produced by** ***Seltsamia galinsogisoli* sp. nov., isolated from *Galinsoga parviflora* as potential inhibitors of FtsZ**

Tian-Yuan Zhang^1+^, Ying-Ying Wu^1+^, Meng-Yue Zhang^1^, Juan Cheng^1^, Blessings Dube^1^, Hui-Jia Yu^1^ and Yi-Xuan Zhang^1^*

^1^School of Life Science and Biopharmaceutics, Shenyang Pharmaceutical University, Shenyang 110016, China.

^+^These authors contributed equally to this work.

*Corresponding authors:

Yi-Xuan Zhang*, School of Life Science and Biopharmaceutics, Shenyang Pharmaceutical University, Shenyang110016, China. E-mail: zhangyxzsh@163.com. Telephone number: +86-24-23986576, Fax number: +86-24-23986401

Table S1. MIC Values of the crude extracts of endophytic Fungi isolated from *Galinsoga parviflora* L…............................................................................................................... 3

[Fig. S1. ^1^H NMR spectrum of **1** (600 MHz, DMSO-d_6_) ................................................4](#__RefHeading___Toc491780349)

[Fig. S2. ^13^C NMR spectrum of **1** (150 MHz, DMSO-d_6_) 5](#__RefHeading___Toc491780350)

[Fig. S3. HSQC spectrum of **1** (600 MHz, DMSO-d_6_) 5](#__RefHeading___Toc491780351)

[Fig. S4. HMBC spectrum of **1** (600 MHz, DMSO-d_6_) 5](#__RefHeading___Toc491780352)

[Fig. S5. HRESIMS spectrum of **1** 6](#__RefHeading___Toc491780354)

[Fig. S6. ^1^H NMR spectrum of **2** (600 MHz, DMSO-d_6_) 6](#__RefHeading___Toc491780354)

[Fig. S7. ^13^C NMR spectrum of **2** (150 MHz, DMSO-d_6_) 6](#__RefHeading___Toc491780356)

[Fig. S8. HSQC spectrum of **2** (600 MHz, DMSO-d_6_) 7](#__RefHeading___Toc491780358)

[Fig. S9. HMBC spectrum of **2** (600 MHz, DMSO-d_6_) 7](#__RefHeading___Toc491780359)

[Fig. S10. HRESIMS spectrum of **2**. 7](#__RefHeading___Toc491780361)

**Table. S1 Antibacterial effect of the crude extracts of endophytic fungi isolated from *Galinsoga parviflora*.**

| No. | Closest known relative and accession number | ident. % | 1 | 2 | 3 | 4 | 5 |
| --- | --- | --- | --- | --- | --- | --- | --- |
| SYP-F-7304 | *Acremonium persicinum* | 99% | ++ | + | + | + | + |
| SYP-F-7295 | *Alternaria alternata* | 99% | - | - | - | - | - |
| SYP-F-7289 | *Alternaria arborescens* | 99% | - | - | - | - | - |
| SYP-F-7288 | *Alternaria tenuissima* | 99% | - | - | - | - | - |
| SYP-F-7345 | *Beauveria bassiana* | 99% | + | ++ | + | + | + |
| SYP-F-7347 | *Chaetopyrena penicillata* | 99% | - | - | - | - | - |
| SYP-F-7353 | *Cladosporium cladosporioides* | 99% | + | + | + | ++ | + |
| SYP-F-7292 | *Cladosporium tenuissimum* | 99% | - | - | - | - | - |
| SYP-F-7338 | *Discosia pseudoartocreas* | 98% | - | - | - | - | - |
| SYP-F-7297 | *Fusarium acuminatum* | 98% | - | - | - | - | - |
| SYP-F-7299 | *Fusarium avenaceum* | 99% | - | - | - | - | - |
| SYP-F-7293 | *Fusarium equiseti* | 99% | - | - | - | - | - |
| SYP-F-7300 | *Fusarium lateritium* | 99% | - | - | - | - | - |
| SYP-F-7348 | *Fusarium solani* | 99% | - | - | - | - | - |
| SYP-F-7298 | *Gibberella avenacea* | 99% | - | - | - | - | - |
| SYP-F7336 | ***Pyrenochaeta sp.*** | 93% | +++ | ++ | ++ | ++ | ++ |
| SYP-F-7303 | *Paraphoma chrysanthemicola* | 99% | - | - | - | - | - |
| SYP-F-7354 | *Penicillium raistrickii* | 99% | + | ++ | + | + | + |
| SYP-F-7337 | *Periconia macrospinosa* | 96% | - | - | - | - | - |
| SYP-F-7342 | *Peyronellaea glomerata* | 99% | - | - | - | - | - |
| SYP-F-7290 | *Phoma eupyrena* | 99% | - | - | - | - | - |
| SYP-F-7301 | *Phoma exigua* | 99% | - | - | - | - | - |
| SYP-F-7349 | *Phoma medicaginis* | 99% | ++ | + | + | + | + |
| SYP-F-7339 | *Scolecobasidium humicola* | 99% | + | ++ | + | + | + |
| SYP-F-7341 | *Sporobolomyces lactosus* | 99% | - | - | - | - | - |
| SYP-F-7351 | *Trichoderma atroviride* | 99% | + | + | + | + | - |
| SYP-F-7757 | *Alternaria alternata* | 99% | - | - | - | - | - |
| SYP-F-7754 | *Botryotinia fuckeliana* | 99% | - | - | - | - | - |
| SYP-F-7734 | *Cadophora orchidicola* | 99% | - | - | - | - | - |
| SYP-F-7799 | *Cladosporium perangustum* | 99% | - | - | - | - | - |
| SYP-F-7781 | *Cladosporium tenuissimum* | 99% | - | - | - | - | - |
| SYP-F-7845 | *Clonostachys rosea* | 99% | - | - | - | - | - |
| SYP-F-7830 | *Cylindrocarpon pauciseptatum* | 99% | + | - | - | - | + |
| SYP-F-7773 | *Epicoccum nigrum* | 99% | + | + | - | - | + |
| SYP-F-7818 | *Fusarium commune* | 99% | + | + | - | - | + |
| SYP-F-7724 | *Fusarium oxysporum* | 99% | - | - | - | - | - |
| SYP-F-7811 | *Fusarium solani* | 99% | - | - | - | - | - |
| SYP-F-7740 | *Fusarium sporotrichioides* | 99% | - | - | - | - | - |
| SYP-F-7764 | *Mucor nederlandicus* | 98% | - | - | - | - | - |
| SYP-F-7730 | *Paraphoma chrysanthemicola* | 99% | - | - | - | - | - |
| SYP-F-7767 | *Penicillium camemberti* | 99% | - | - | - | - | - |
| SYP-F-7775 | *Penicillium manginii* | 99% | - | - | - | - | - |
| SYP-F-7742 | *Phialophora mustea* | 98% | - | - | - | - | - |
| SYP-F-7840 | *Phoma eupyrena* | 99% | - | - | - | - | - |
| SYP-F-7741 | *Phomopsis columnaris* | 99% | - | - | - | - | - |
| SYP-F-7723 | *Plectosphaerella cucumerina* | 99% | - | - | - | - | - |
| SYP-F-7732 | *Sarocladium strictum* | 99% | - | - | - | - | - |
| SYP-F-7728 | *Talaromyces sp.* | 99% | - | - | - | - | - |
| SYP-F-7737 | *Volutella ciliata* | 99% | - | - | - | - | - |

1: *Staphylococcus aureus,* 2: *Bacillus subtillis,* 3: *Pseudomonas aeruginosa,* 4: *Klebsiella pneumonia, 5: Bacillus cereus*“+”：antibacterial rate 0-30%，“++”：30-60%，“+++”60-80%，“++++”80-100%，“-”no antibacterial activities.

**
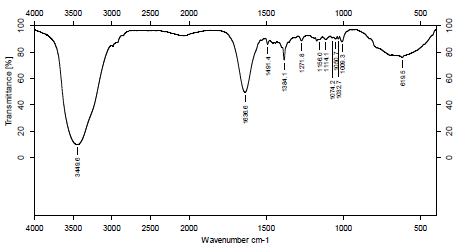
**

# Fig. S1 IR spectrum of 1

**
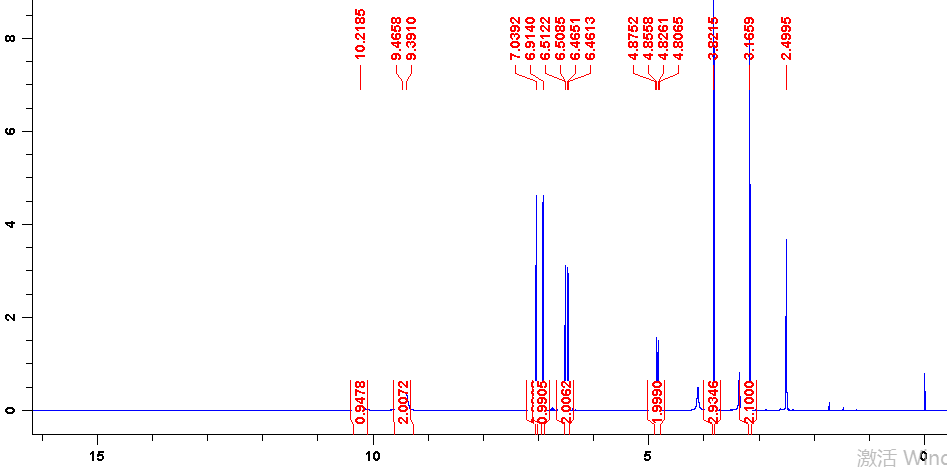
**

# Fig. S2 ^1^H NMR spectrum of 1 (600 MHz, DMSO-d_6_)


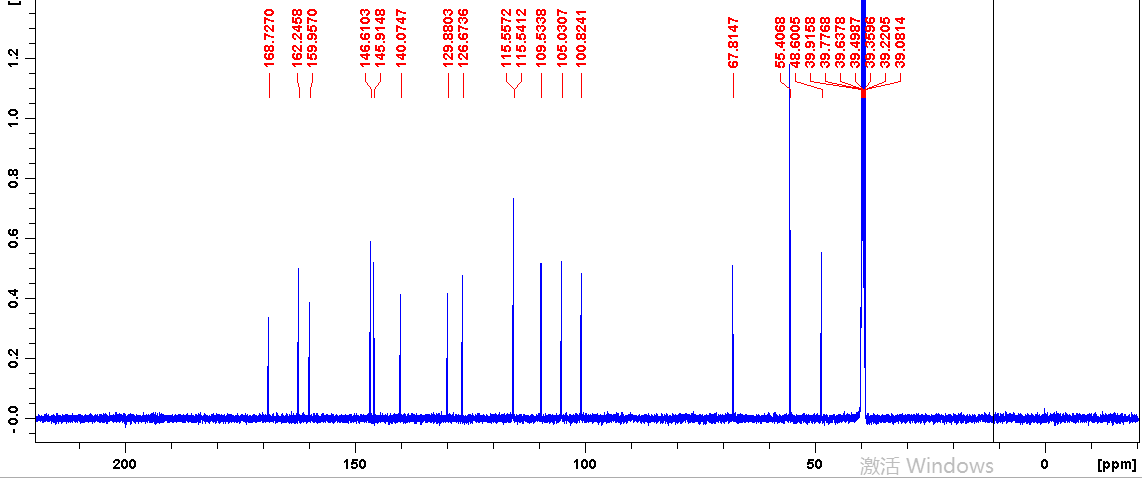


# Fig. S3 ^13^C NMR spectrum of 1 (150 MHz, DMSO-d_6_)


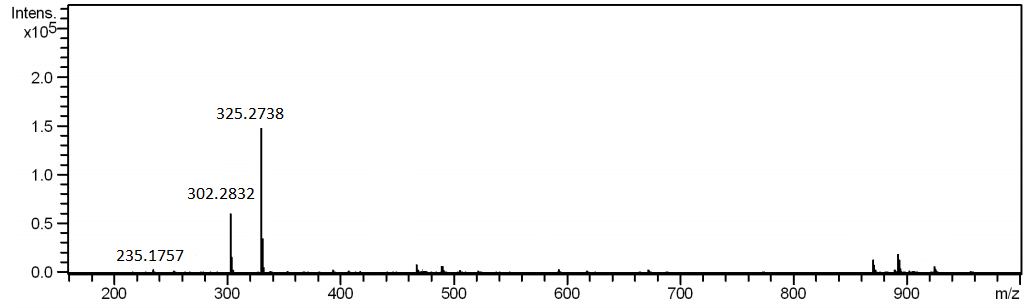


**Fig. S4 MS spectrum of 1**

**
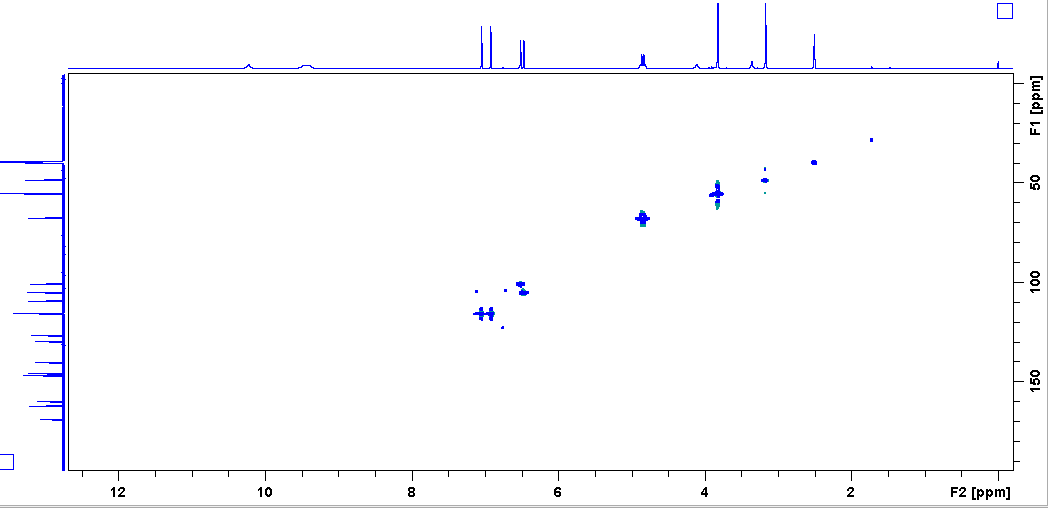
**

# Fig. S5 HSQC spectrum of 1 (600 MHz, DMSO-d_6_)

**
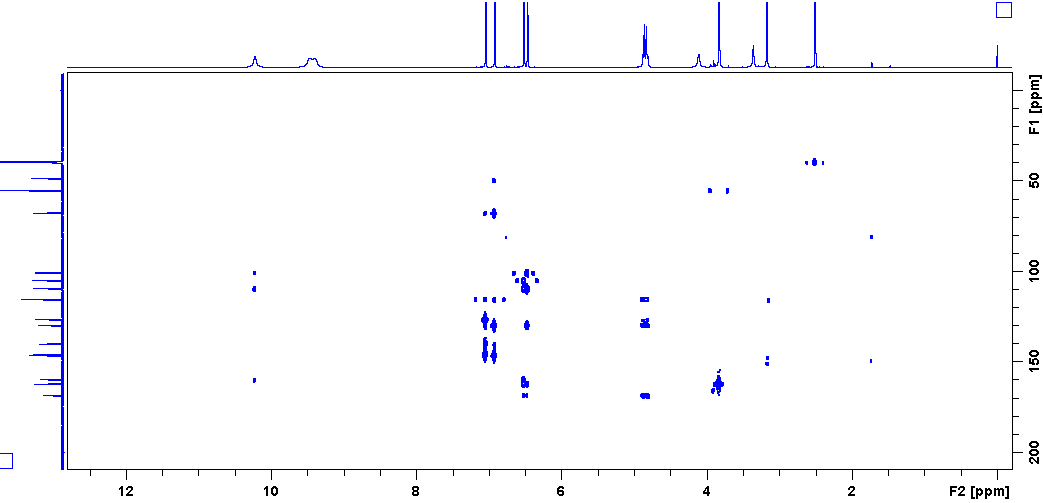
**

# Fig. S6 HMBC spectrum of 1 (600 MHz, DMSO-d_6_)

#
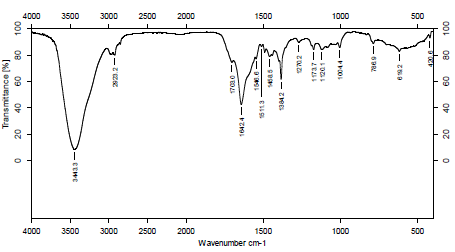


#

# Fig. S7 IR spectrum of 2

**
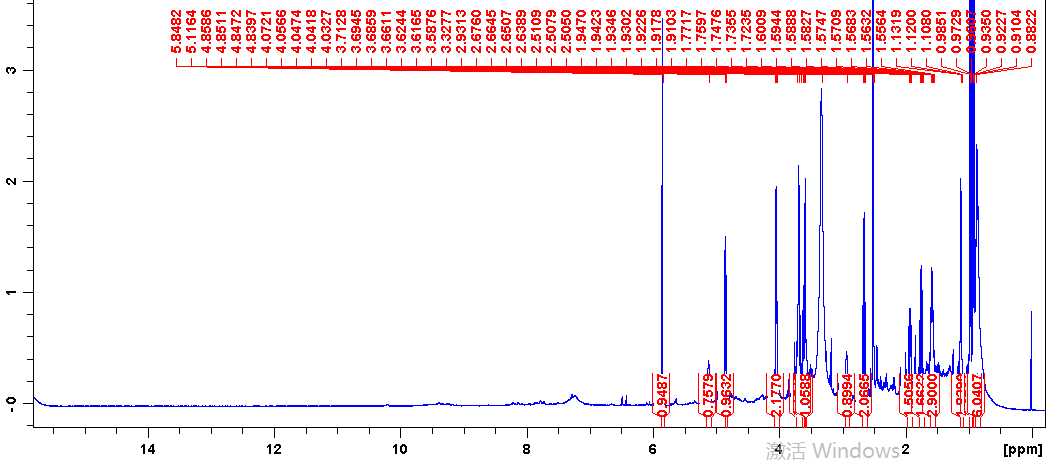
**

**Fig. S8 ^1^H NMR spectrum of 2 (600 MHz, DMSO-*d*_6_)**


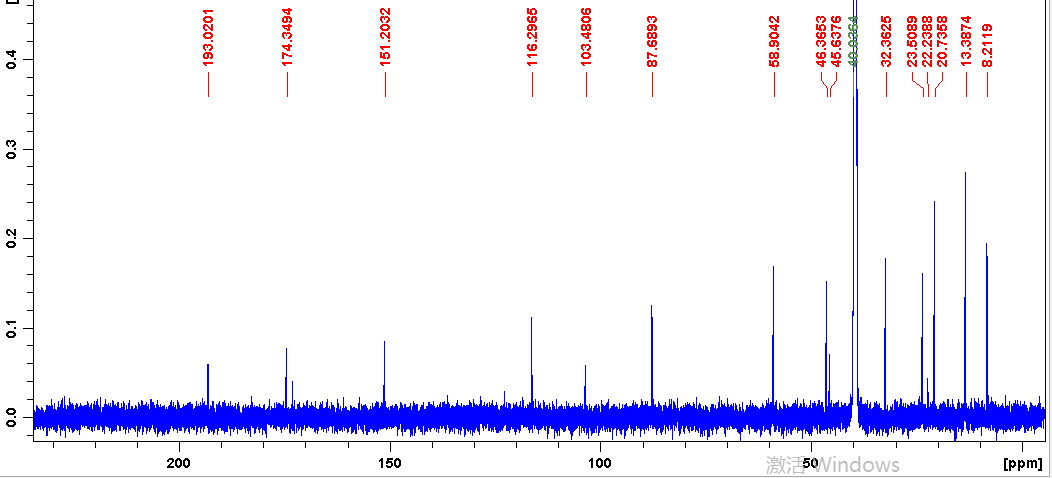


# Fig. S9 ^13^C NMR spectrum of 2 (150 MHz, DMSO-*d*_6_)


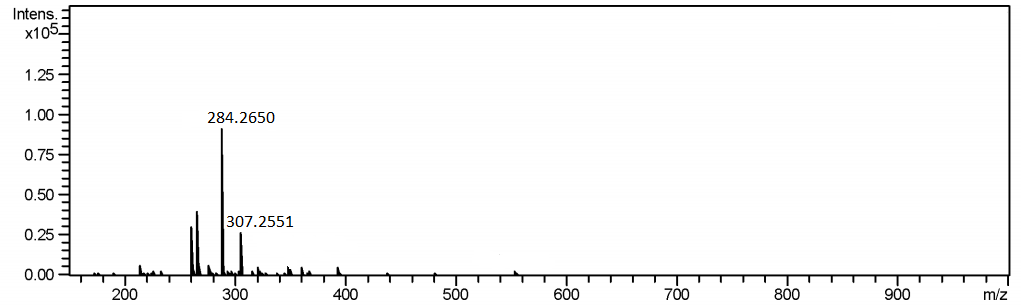


**Fig. S10 MS spectrum of 2**


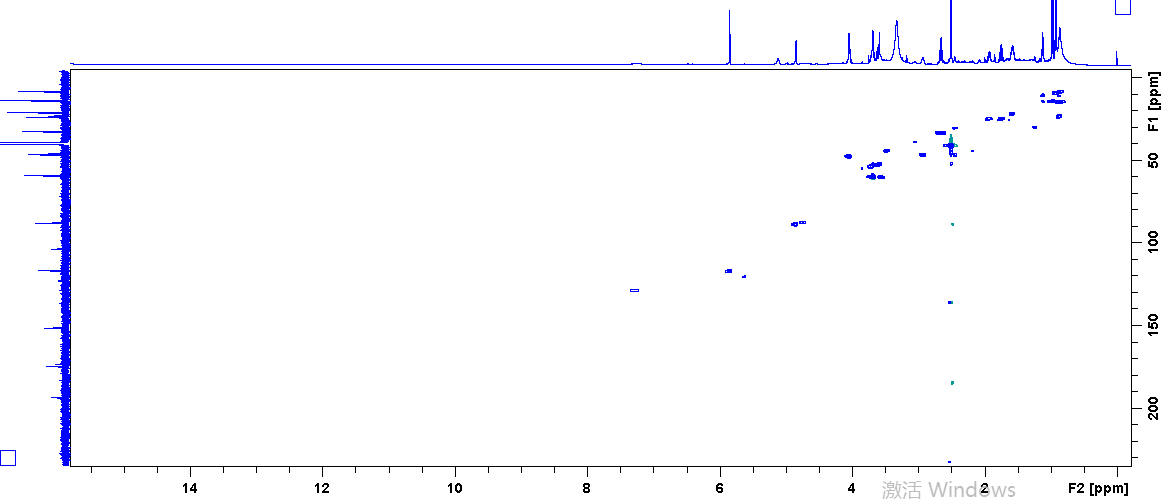


**Fig. S11 HSQC spectrum of 2 (600 MHz, DMSO-*d*_6_)**

**
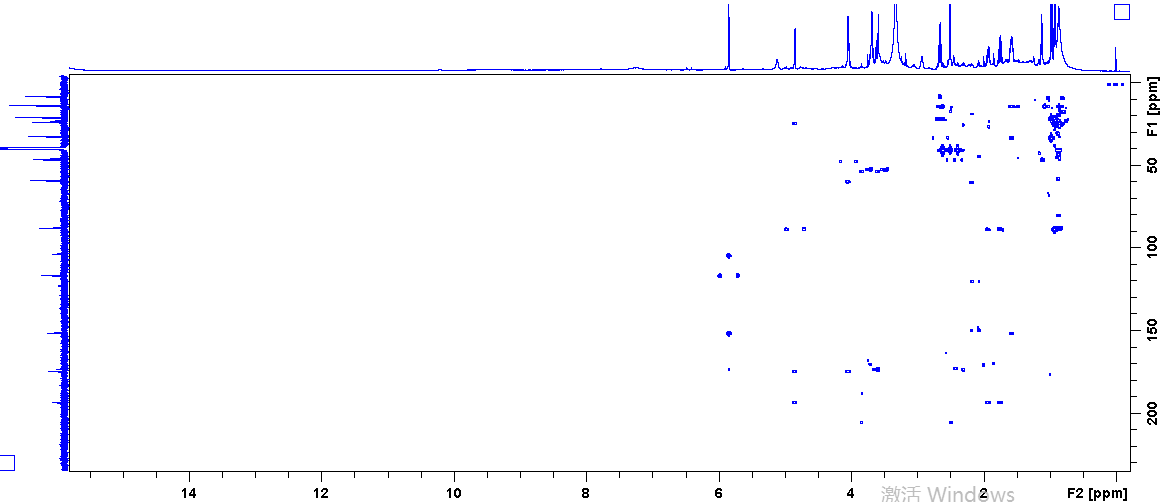
**

# Fig. S12 HMBC spectrum of 2 (600 MHz, DMSO-*d*_6_)
